# Risk factors for death of trauma patients admitted to an Intensive Care Unit*

**DOI:** 10.1590/1518-8345.3482.3236

**Published:** 2020-02-14

**Authors:** Maicon Henrique Lentsck, Rosana Rosseto de Oliveira, Ligiana Pires Corona, Thais Aidar de Freitas Mathias

**Affiliations:** 1 Universidade Estadual de Maringá, Departamento de Enfermagem, Maringá, PR, Brazil.; 2 Universidade Estadual do Centro-Oeste, Departamento de Enfermagem, Guarapuava, PR, Brazil.; 3 Scholarship holder at the Coordenação de Aperfeiçoamento de Pessoal de Nível Superior (CAPES), Brazil.; 4 Universidade Estadual de Campinas, Faculdade de Ciências Aplicadas, Campinas, SP, Brazil.

**Keywords:** Wounds and Injuries, Injuries, Intensive Care Unit, Critical Care, Death, Risk Factors, Feridas e Lesões, Ferimentos, Unidade de Terapia Intensiva, Cuidados Intensivos, Óbito, Fatores de Risco, Heridas y Lesiones, Lesiones, Unidad de Terapia Intensiva, Cuidados Intensivos, Fallecimiento, Factores de Riesgo

## Abstract

**Objective:**

To analyze the risk factors for death of trauma patients admitted to the intensive care unit (ICU).

**Method:**

Retrospective cohort study with data from medical records of adults hospitalized for trauma in a general intensive care unit. We included patients 18 years of age and older and admitted for injuries. The variables were grouped into levels in a hierarchical manner. The distal level included sociodemographic variables, hospitalization, cause of trauma and comorbidities; the intermediate, the characteristics of trauma and prehospital care; the proximal, the variables of prognostic indices, intensive admission, procedures and complications. Multiple logistic regression analysis was performed.

**Results:**

The risk factors associated with death at the distal level were age 60 years or older and comorbidities; at intermediate level, severity of trauma and proximal level, severe circulatory complications, vasoactive drug use, mechanical ventilation, renal dysfunction, failure to perform blood culture on admission and Acute Physiology and Chronic Health Evaluation II.

**Conclusion:**

The identified factors are useful to compose a clinical profile and to plan intensive care to avoid complications and deaths of traumatized patients.

## Abstract (pt)

**Objetivo:**

analisar os fatores de risco para óbito de pacientes com trauma internados em Unidade de Terapia Intensiva (UTI).

**Método:**

estudo de coorte retrospectivo, com dados de prontuários de adultos hospitalizados por trauma em Unidade de Terapia Intensiva geral. Foram incluídos pacientes de 18 anos ou mais de idade e admitidos por lesões. As variáveis foram agrupadas em níveis de maneira hierarquizada. O nível distal contemplou variáveis sociodemográficas, da internação, causa do trauma e comorbidades; o intermediário, as características do trauma e do atendimento pré-hospitalar; o proximal, as variáveis dos índices prognósticos, da admissão intensiva, procedimentos e complicações. Realizou-se análise de regressão logística múltipla.

**Resultados:**

os fatores de risco associados ao óbito no nível distal foram idade igual ou superior a 60 anos e comorbidades; no nível intermediário, a gravidade do trauma e no nível proximal, as complicações circulatórias graves, uso de drogas vasoativas, ventilação mecânica, disfunção renal, não realização de hemocultura na admissão e *Acute Physiology and Chronic Health Evaluation II*. **Conclusão:** os fatores identificados são úteis para compor um perfil clínico e para planejar a assistência intensiva a fim de evitar complicações e óbitos de pacientes traumatizados.

## Abstract (es)

**Objetivo:**

analizar los factores de riesgo para muerte de pacientes con trauma internados en unidad de terapia intensiva.

**Método:**

estudio de cohorte retrospectivo, con datos de fichas médicas de adultos hospitalizados por trauma en unidad de terapia intensiva general. Fueron incluidos pacientes de 18 años o más de edad y admitidos por lesiones. Las variables fueron agrupadas en niveles de manera jerarquizada. El nivel distal contempló variables sociodemográficas, internación, causa del trauma, y comorbilidades; el nivel intermedio las características del trauma y de la atención prehospitalaria; el nivel proximal las variables de índices pronósticos, de admisión intensiva, de procedimientos y complicaciones. Se realizó análisis de regresión logística múltiple.

**Resultados:**

los factores de riesgo asociados a la muerte en el nivel distal fueron: edad igual o superior a 60 años y comorbilidades; en el nivel intermedio la gravedad del trauma; y, en el nivel proximal las complicaciones circulatorias graves, uso de drogas vaso activas, ventilación mecánica, disfunción renal, no realización de hemocultivo en la admisión y Acute Physiology and Chronic Health Evaluation II.

**Conclusión:**

los factores identificados son útiles para componer un perfil clínico y para planificar la asistencia intensiva con la finalidad de evitar complicaciones y muertes de pacientes traumatizados.

## Introduction

Trauma has become international concern due to thousands of deaths^(^ 1 ^)^. In 2013 alone, about 4.8 million people died from an injury^(^ 2 ^)^. In Brazil, trauma accounts for 12.4% of all deaths and is the leading cause of death among young people under 44 years of age^(^ 3 ^)^, and is heterogeneous in its causes, types of injuries, severity and risk factors. This heterogeneity influences the clinical prognosis and requires the use of a comprehensive care system, which in turn depends on different clinical and surgical structures, organizations and specialties^(^ 4 ^)^.

Trauma management requires a multidisciplinary approach that begins at the trauma site where prehospital care (PHC) plays a key role. After this phase, patient stabilization assistance should be performed in an outpatient or equipped hospital setting. The patient will be kept under observation in a hospital environment, especially intensive, which prioritizes the care of the most life-threatening traumatized^(^ 5 ^)^.

In order to know the profile of morbidity and mortality due to trauma, most research explores, mainly, APH information and data^(^ 6 ^-^ 7 ^)^, even if this condition requires hospitalization and intensive care units (ICU)^(^ 8 ^)^. After the initial trauma care, either by accident or violence, in the period between the occurrence of trauma, hospital admission and the ICU stay, several factors remain associated with the death of the most severe cases. Early identification of factors associated with death from ICU trauma, which consider severity and trauma care at all stages of care, may have a positive impact on patient prognosis^(^ 5 ^)^.

As already identified, the increase in teams and the quality of PHC; environmental and vehicular safety^(^ 9 ^-^ 10 ^)^; improvement of techniques and diagnostics such as computed tomography, patient management and treatment strategies; the use of transfusion protocols using plasma, platelets, red blood cell concentrate and tranexamic acid^(^ 11 ^)^ and integrated cooperation between the components of the trauma care system and the prevention of trauma among the population^(^ 10 ^))^ are strategies that have been aimed at reducing the mortality of trauma victims admitted to the ICU. 

The objective of this study, considering the increase of accidents and violence in Brazil that directly impact the demand for specialized hospital beds, to analyze the risk factors for the death of trauma patients admitted to the ICU. This study was guided by the hypothesis that characteristics of the individual, type and severity of trauma, prehospital care and hospital care are associated with death. Therefore, analyzing the determinants of death in traumatized ICU patients becomes especially important when these determinants are organized in a hierarchical manner at different levels of determination. It is believed that this analysis strategy can contribute to the knowledge of factors associated with death and, above all, to monitor and direct patient care with the adoption of qualified interdisciplinary and inter-professional care.

## Method

Retrospective cohort study with data from medical records of adult individuals, 18 years of age and over, hospitalized for trauma in ICU. The ICU under study is general, with ten beds, in a referral hospital for approximately 500,000 inhabitants located in the mesoregion of the center of southern Paraná^(^ 12 ^)^.

A total of 569 hospitalizations from January 1, 2013 to December 31, 2016 were selected and identified in the admission book with reasons for hospitalization of any injury due to external cause. The initial selection criteria were hospitalizations with mention of trauma, external cause and procedure related to trauma care. After analysis of each hospitalization, the study excluded hospitalizations related to procedures not related to trauma management (101), with incomplete records (31) and occurring in children under 18 years (9). Trauma related to burns (3) and poisoning (8) was also excluded, in order to make the sample homogeneous, since these are considered specific types of trauma, requiring differentiated intensive care. The study sample totaled 417 individuals. 

Data was collected primarily in the electronic medical records in consultation with all documents such as clinical evolution, medical and nursing prescriptions, control and annotations of procedures, results of laboratory and imaging tests, prehospital and Hospital Infection Control Service records (HICS). In addition, the physical medical record was accessed.

The determinants for ICU trauma death were classified into three levels: distal, intermediate and proximal. The insertion of the possible determinants in the levels followed an order previously established by a theoretical model defined “a priori”, based on the literature and on possible relationships^(^ 5 ^,^ 13 ^)^ It is considered a strategy to deal with all conceptually related variables and, therefore, has the potential to assist in the identification and analysis of risk factors.

Distal level refers to variables that are farthest from the outcome and act indirectly through proximal determinants to affect the risk of death from trauma. At this level, the following variables were considered available in the chart: sociodemographic^(^ 14 ^)^ (gender, age, place of residence); of hospitalization (type of financing, day of week and time of admission); cause of trauma^(^ 15 ^)^ (work accident or suicide attempt) and Charlson Comorbidity Index - (CCI)^(^ 13 ^-^ 14 ^)^. 

The intermediate level contains variables that broaden the understanding of proximal determinants and value the link between trauma information and its assistance before definitive ICU treatment. For this level, the characteristics of the trauma were grouped^(^ 13 ^-^ 14 ^))^ (Revised Trauma Score - RTS, Injury Severity Score - ISS, New Injury Severity Score - NISS; number of affected body regions and most severe body region; type of trauma; sanitary transport and external cause) and prehospital care (PHC)^(^ 5 ^,^ 7 ^)^ (basic and advanced respiratory and circulatory supports, vasoactive drugs and ethyl breath). 

**Figure 1:**
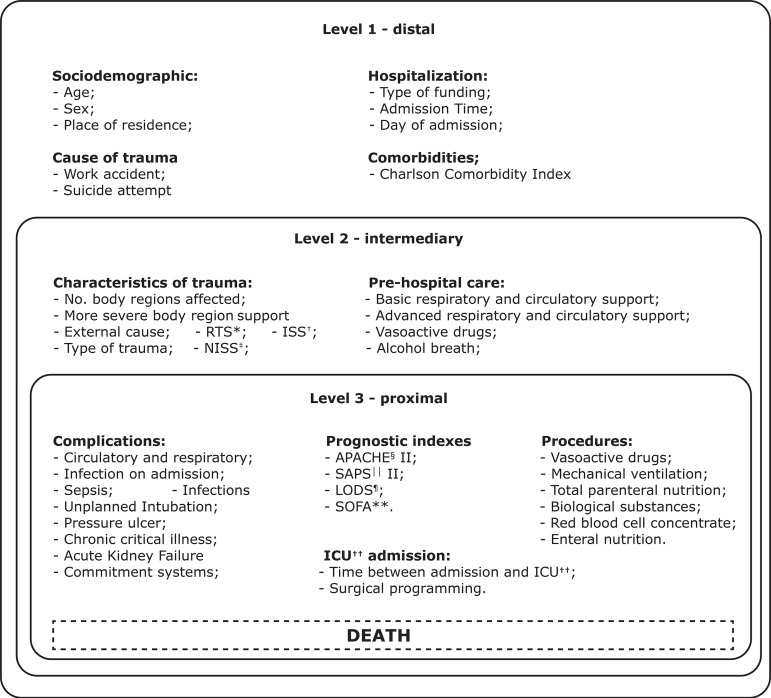
Hierarchical theoretical model for the determination of death in trauma patients hospitalized in ICU^††^. Guarapuava, PR, Brazil, 2018 ^*^RTS = Revised Trauma Score; ^†^ISS = Injury Severity Score; ^‡^NISS = New Injury Severity Score; ^§^APACHE = Acute Physiology and Chronic Health Evaluation; ^||^SAPS = Simplified Acute Physiology Score; ^¶^LODS = Logistic Organ Dysfunction System; ^**^SOFA = Sepsis = Related Organ Failure Score; ^††^ICU – Intensive Care Unit

The proximal level consisted of determinants closely linked to death by trauma and organized into groups of variables: prognostic indices measured within the first 24 hours^(^ 16 ^)^ (Acute Physiology And Chronic Health Evaluation - APACHE II; Simplified Acute Physiology Score - SAPS II; Logistic Organ Dysfunction System - LODS II; Sepsis-Related Organ Failure Score - SOFA); ICU procedures^(^ 17 ^-^ 18 ^)^ (administration of biological substances; red blood cell concentrate within the first 24 hours; enteral nutrition; total parenteral nutrition; vasoactive drugs and use of mechanical ventilation); characteristics of ICU admission^(^ 17 ^)^ (time interval between admission and ICU and surgical programming) and complications during ICU stay^(^ 19 ^)^ (Figure 1). 

The following were considered ICU complications: severe circulatory (cardiopulmonary arrest - CPA, deep venous thrombosis and acute myocardial infarction - AMI); severe respiratory disorders (pulmonary embolism and acute respiratory distress syndrome - ARDS); cardiological, hematological, hepatic, neurological, renal and pulmonary dysfunctions measured by LODS within the first 24 hours; renal failure during hospitalization; unplanned intubation; infections (pulmonary, bloodstream, urinary tract and surgical site); pressure ulcer; sepsis; infection on admission and chronic intensive hospitalization (Chronic Critical Disease - CCD).

Multiple logistic regression analysis was performed with hierarchical entry of the variables, in levels, in the following order: the distal ones, which condition all the others; the intermediate conditions, which condition those of the lower level, and the proximal ones, which directly predict death (Figure 1). This analysis is used to explain the relationship between variables in models whose set of empirical propositions already indicates the strength and direction of the relationship and allows to identify whether the association is direct or mediated by the effect of other variables^(^ 20 ^)^.

The multiple logistic regression model, with the inclusion of the stepwise forward variables, considered those with p-value <0.20 in the univariate analysis, and the variables with p <0.05 or that fit the model remained in the final model. The magnitude of the associations was estimated by Odds Ratio (OR), with 95% confidence intervals as a measure of precision. The adequacy of the final model was verified from the deviance tests, Hosmer-Lemeshow, and the collinearity of the variables was tested with the variance inflation factor (VIF). Statistical analysis was performed using Stata 12.0 software. 

The presentation of the models followed the steps of insertion of the variables of each level. Model A shows associations of sociodemographic factors (level 1) and death; model B shows associations of sociodemographic factors, trauma characteristics and PHC and death (levels 1 and 2) and model C shows associations of sociodemographic factors, trauma characteristics and PHC and characteristics of ICU care (levels 1, 2 and 3) and death, with its respective adjustments. The study was approved by the Research Ethics Committee Involving Human Beings of the State University of Maringá (REC/UEM protocol nº 1.835.356 / 2016).

## Results

The ICU trauma mortality rate was 28.2%. Tables 1, 2 and 3 show the univariate analyzes with the associations included in the multiple model (p <0.20). Death was associated with age from 40 to 59 years and 60 years and over, Charlson’s comorbidity index (distal level variables) and penetrating trauma, falls, severity of trauma (estimated by RTS, ISS and NISS), procedures performed at the time of prehospital care (advanced respiratory support, advanced circulatory support) and the presence of ethyl breath (intermediate level variables) (Table 1).

**Table 1:** Univariate analysis of the association of the variables distal, intermediate levels and death from trauma in ICU admissions* (n-417). Guarapuava, PR, Brazil, 2018

Variables	Death		Discharge			*P* value
	n	%	N	%	OR^†^	
Level 1 - distal						
Age						
18 to 39	58	49,2	211	70,6		Ref^‡^.
40 to 59	35	29,7	65	21,7	1,95	0,009
60 and more	25	21,2	23	7,7	3,95	<0,001
Type of funding						
UHS^§^	114	96,6	278	93,0	2,15	0,168
Not UHS^§^	4	3,4	21	7,0		Ref^‡^.
CCI^||^ (mean and standard deviation)	0,9	1,9	0,2	1,0	1,35	<0,001
Level 2 - intermediary						
External cause						
Agressions	19	16,1	79	26,4		Ref^‡^.
Traffic accidents	71	60,2	183	61,2	1,61	0,101
Falls	23	19,5	29	9,7	3,29	0,002
Other external causes	5	4,2	8	2,7	2,59	0,126
Type of trauma						
Blunt	102	86,4	243	81,3		Ref^‡^.
Penetrating	16	13,6	56	18,7	1,46	0,210
Advanced Respiratory Support						
No	31	26,3	36	12,0		Ref^‡^.
Yes	87	73,7	263	88,0	0,38	<0,001
Advanced Circulatory Support						
No	82	69,5	164	54,8		Ref^‡^.
Yes	36	30,5	135	45,2	0,53	0,007
Alcohol breath						
No	108	91,5	249	83,3		Ref^‡^.
Yes	10	8,5	50	16,7	0,46	0,034
Vasoactive drugs						
No	113	95,8	295	98,7		Ref^‡^.
Yes	5	4,2	4	1,3	3,26	0,082
RTS^¶^ (mean and standard deviation)	9,2	2,2	10,5	1,8	0,72	<0,001
ISS** (mean and standard deviation)	21,0	8,6	15,8	8,2	1,07	<0,001
NISS^††^ (mean and standard deviation)	27,8	11,9	21,0	12,2	1,04	<0,001

* ICU = Intensive Care Unit;

† OR = Odds Ratio;

‡ Ref. = Reference;

§ SUS = Unified Health System;

|| ICC = Charlson Comorbidity Index;

¶ RTS = Revised Trauma Score;

** ISS = Injury Severity Score;

†† NISS = New Injury Severity Score

**Table 2:** Univariate analysis of association of variables, procedures and prognostic indices of proximal level and death from trauma in ICU admissions* (n-417). Guarapuava, PR, Brazil, 2018

Level 3 - proximal	Death		Discharge			P value
	n	%	n	%	OR^†^	
ICU Procedures*						
Vasoactive drugs						
No	36	30,5	253	84,6		Ref^‡^.
Yes	82	69,5	46	15,4	12,52	<0,001
Mechanical ventilation						
No	11	9,3	157	52,5		Ref^‡^.
Yes	107	90,7	142	47,5	10,75	<0,001
Total parenteral nutrition						
No	106	89,8	294	98,3		Ref^‡^.
Yes	12	10,2	5	1,7	6,65	<0,001
Biological substances						
No	39	33,1	157	52,5		Ref^‡^.
Yes	79	66,9	142	47,5	2,23	<0,001
Red blood cell concentrate						
No	66	55,9	220	73,6		Ref^‡^.
Yes	52	44,1	79	26,4	2,19	0,001
Enteral Nutrition						
No	77	65,3	224	74,9		Ref^‡^.
Yes	41	34,7	75	25,1	1,59	0,048
Prognostic Indexes						
APACHE^§^ II (mean and standard deviation)	17,9	7,7	10,0	6,0	1,17	<0,001
SAPS^||^ II (mean and standard deviation)	41,9	15,8	24,4	14,1	1,07	<0,001
LODS^¶^ (mean and standard deviation)	6,7	3,7	3,5	2,9	1,33	<0,001
SOFA** (mean and standard deviation)	5,7	3,0	2,8	2,5	1,42	<0,001

* ICU = Intensive Care Unit;

† OR = Odds Ratio;

‡ Ref. = Reference;

§ APACHE = Acute Physiology and Chronic Health Evaluation;

|| SAPS = Simplified Acute Physiology Score;

¶ ODS = Logistic Organ Dysfunction System;

** SOFA = Sepsis-Related Organ Failure Score

**Table 3:** Univariate analysis of the association of proximal level complications and death from trauma in ICU admissions* (n-417). Guarapuava, PR, Brazil, 2018

Level 3 - proximal	Death		Discharge			*P value*
	N	%	n	%	OR^†^	
ICU Complications*						
Severe circulatory complications						
No	90	76,3	292	97,7		Ref^‡^.
Yes	28	23,7	7	2,3	12,97	<0,001
Severe respiratory complications						
No	106	89,8	292	97,7		Ref^‡^.
Yes	12	10,2	7	2,3	4,72	0,001
Acute Kidney Failure						
No	103	87,3	293	98,0		Ref^‡^.
Yes	15	12,7	6	2,0	2,60	<0,001
Pulmonary dysfunction						
No	41	34,7	208	69,6		Ref^‡^.
Yes	77	65,3	91	30,4	4,29	<0,001
Renal dysfunction						
No	49	41,5	194	64,9		Ref^‡^.
Yes	69	58,5	105	35,1	2,60	<0,001
Neurological dysfunction						
No	30	25,4	122	40,8		Ref^‡^.
Yes	88	74,6	177	59,2	2,02	0,004
Cardiological dysfunction						
No	82	69,5	241	80,6		Ref^‡^.
Yes	36	30,5	58	19,4	1,82	0,015
Hepatic dysfunction						
No	48	40,7	151	50,5		Ref^‡^.
Yes	70	59,3	148	49,5	1,48	0,071
Sepsis						
No	110	93,2	294	98,3		Ref^‡^.
Yes	8	6,8	5	1,7	4,27	0,012
Unplanned Intubation						
No	95	80,5	273	91,3		Ref^‡^.
Yes	23	19,5	26	8,7	2,54	0,003
Pressure ulcer						
No	100	84,7	274	91,6		Ref^‡^.
Yes	18	15,3	25	8,4	1,97	0,040
Chronic critical illness						
No	101	85,6	275	92,0		Ref^‡^.
Yes	17	14,4	24	8,0	1,92	0,052
Infection on admission						
No	65	55,1	188	62,9		Ref^‡^.
Yes	5	4,2	9	3,0	1,60	0,410
Exam not carried out	48	40,7	102	34,1	1,36	0,174

* ICU = Intensive Care Unit;

† OR = Odds Ratio;

‡ Ref. = Reference

Among the proximal level variables, death was associated with patient severity indexes (APACHE II, SAPS II, LODS and SOFA), vasoactive drugs, mechanical ventilation, total parenteral nutrition, biological substances, red blood cell concentrate and enteral nutrition (Table 2). 

Of the complications in the ICU, severe circulatory and respiratory, renal, pulmonary, neurological, cardiac and hepatic dysfunction, sepsis, pressure injury and unplanned intubation were associated with death (Table 3).

In model A of the hierarchical multiple regression analysis, age and comorbidities (CCI) remained independently associated with death from trauma in the ICU. In the presence of intermediate level 2 variables (model B), comorbidities lost significance and the association of death with age and the ISS (trauma severity index) remained. In the last stage of the analysis, with the presence of the proximal level variables, the independent risk factors for death aged 60 years or older, comorbidities (CCI) and trauma severity (ISS) (model C) remained as independent risk factors. Of the proximal level variables, severe circulatory complications, use of vasoactive drugs, mechanical ventilation, renal dysfunction, failure to perform the infection detection examination at ICU admission and APACHE II (patient severity) remained associated with death. Circulatory complications were highlighted, with OR-7.33 (IC-2.43; 22.06), the use of mechanical ventilation, with OR-5.58 (IC-1.94; 15.98), and vasoactive drugs, with OR-5.09, in addition to the age of 60 years or older, with OR-3.77 (IC-1.03; 13.82) (Table 4). 

**Table 4:** Multiple logistic regression analysis for trauma death and risk factors in ICU patients* (n-417). Guarapuava, PR, Brazil, 2018

Independent variable	Model not adjusted		Model A		Model B		Model C	
	OR^†^	CI^‡^ 95%	OR^†^	CI^‡^ 95%	OR^†^	CI^‡^ 95%	OR^†^	CI^‡^ 95%
Level 1 - distal^§^								
Age								
40 to 59	1,95	1,18; 3,24	1,85	1,11; 3,08	2,00	1,14; 3,52	1,40	0,69; 2,86
60 and more	3,95	2,09; 7,47	2,23	0,93; 5,37	3,98	1,51; 10,50	3,77	1,03; 13,82
CCI||	1,35	1,16; 1,57	1,21	0,99; 1,48	1,20	0,97; 1,49	1,41	1,03; 1,94
Level 2 - intermediary^¶^								
Advanced Circulatory Support	0,53	0,34; 0,84			0,37	0,19; 0,71	0,71	0,36; 1,38
Alcohol breath	0,46	0,22; 0,94			0,47	0,21; 1,04	0,41	0,15; 1,12
ISS**	1,07	1,04; 1,10			1,07	1,04; 1,10	1,04	1,00; 1,08
Level 3 - proximal^††^								
Circulatory Complications	12,97	5,49; 30,70					7,33	2,43; 22,06
Vasoactive drugs	12,52	7,58; 20,70					5,09	2,58; 10,04
Mechanical ventilation	10,75	5,55; 20,82					5,58	1,94; 15,98
Renal dysfunction	2,60	1,68; 4,02					2,25	1,21; 4,19
Infection on admission								
Yes	1,60	0,51; 4,96					1,78	0,36; 8,60
Exam not carried out	1,36	0,87; 2,12					2,97	1,50; 5,86
APACHE^‡‡^ II	1,17	6,37; 9,50					1,07	1,02; 1,13

* ICU = Intensive Care Unit;

† OR = Odds Ratio;

‡ 95% CI = 95% Confidence Interval;

§ Level 1 - distal = - ICC adjusted model;

|| ICC = Charlson Comorbidity Index;

¶ Level 2 = Intermediate = ISS adjusted model;

** ISS = Injury Severity Scale;

†† Level 3 = proximal = Model adjusted by APACHE II;

‡‡ APACHE = Acute Physiology and Chronic Health Evaluation

## Discussion

Severe trauma is a worldwide pandemic and a leading cause of death^(^ 2 ^)^. The identification of risk factors for death from trauma in the ICU through hierarchical analysis can aggregate information, especially when many factors are considered in the analysis. This study identified a traumatic ICU mortality rate of 28.2%, which was considered high compared to data from a multicenter study in the US that analyzed 1.03 million adult trauma patients admitted to the ICU in 2013^(^ 19 ^)^. In two regions of Estonia, the results of hospitalizations for severe trauma in 2013 were compared and a mortality rate of 20.7% was identified ^(^ 21 ^)^. Similar data were recorded in a Brazilian ICU, in Sobral - CE, between 2013 and 2014, which identified a mortality rate of 28.6% in traumatized patients^(^ 8 ^)^.

The determinants for death from trauma in the ICU observed in this study were some existing at the time of trauma, such as age over 60 years and comorbidities, the severity of trauma identified from prehospital and emergency care and factors identified during ICU admission, such as the use of mechanical ventilation, renal dysfunction in the first 24 hours and patient severity (APACHE II), as well as vasoactive drugs, circulatory complications and no blood culture on admission.

In this study, age 60 years and over remained a predictor of death, increasing the risk by three times. Worse prognosis for traumatized elderly compared to younger patients has been constantly presented in the literature^(^ 8 ^-^ 9 ^,^ 22 ^-^ 25 ^)^. This weakness is explained by characteristics of the elderly population that make it more vulnerable, such as comorbidities and the use of medications that impact the physiological response to the injury and complicate treatment and recovery^(^ 26 ^)^. Due to trauma-induced catabolism throughout hospitalization, the elderly often experience progressive loss of mass and skeletal muscle strength^(^ 26 ^)^, as shown by research that analyzed the body composition of elderly in French ICU by computed tomography and identified loss of skeletal muscles and adipose tissue, being higher in those with infections^(^ 27 ^)^. Other pathophysiological conditions of the elderly, such as the reduction of endogenous catecholamines, which limits the response to hemorrhage, the reduction of kidney functional reserve by up to 40% of the glomeruli, and the reduction of lung, bone and immunological functions^(^ 28 ^)^ may impact survival of the elderly with trauma. This propensity to physiological deterioration makes the traumatized elderly one of the most vulnerable population groups and, therefore, in their admission to the ICU, this characteristic should be considered in the care provided.

Care for traumatized older people should consider the impact of aging on specific organ functions and, as a result, may affect interventions^(^ 26 ^)^. In this sense, interdisciplinary care improves quality because it addresses the comorbidities, processes, and outcomes of geriatric syndromes, identifies additional diagnoses, assists in advanced care planning, manages drug changes, and pain management ^(^ 29 ^)^ and identifies early risk factors for death ^(^ 30 ^)^. There are gaps in the development and implementation of treatment protocols for traumatized elderly, lacking guidelines and specialized centers^(^ 26 ^)^. This fact was observed in this study, which points to the need to adopt instruments that facilitate the identification and screening of traumatized elderly patients, from PHC to ICU as a priority^(^ 31 ^)^.

It was also found that with each increase in the CCI score, the risk of death, regardless of age adjustment, increased by 41%. Comorbidities may contribute to negative ICU outcomes, such as the greater possibility of complications^(^ 9 ^,^ 19 ^,^ 23 ^-^ 24 ^)^. In this sense, it is important to adopt a classification system that is capable, in addition to the number of comorbidities, also to consider its severity^(^ 9 ^)^.

Trauma, in contemporary society, is not only related to young people, but rather means a grievance that accompanies man during his life. Considering that the population is aging, with the advance in the management of chronic diseases that gives them a more active life, the elderly with comorbidities use drugs such as antiplatelet agents and anticoagulants, for example. Continued medication may increase the risk of bleeding complications, surgical infections, pneumonia, and other infections that contribute to longer ICU stay^(^ 24 ^)^. 

In line with previous research^(^ 19 ^,^ 22 ^,^ 24 ^-^ 25 ^)^, trauma severity was a risk factor for death and the only independent predictor at the intermediate level of determination. Each increase in the severity index (ISS) score resulted in a 4% increase in the risk of death, as identified in other studies, regardless of the cause of the trauma. A study of patients hospitalized for severe trauma (ISS>15) in South Korea identified a 4% increase in the risk of death with each increase in ISS (25), and a study of elderly patients hospitalized for blunt trauma in Israel identified an increase of 1.08% chance of death^(^ 24 ^)^.

The physiological and anatomical evaluation of traumatized patients is an action performed by the ICU health team to know the severity of the trauma, which contributes to guarantee the quality of care ^(^ 32 ^)^. In this context, the use of ISS can be very useful because, in hospitals, especially in the ICU, there is greater availability of information necessary for their scoring than in PHC or immediately after arrival at the hospital, which makes their prognostic ability more efficient^(^ 33 ^)^.

Identification of ICU complications, in addition to improving care practices^(^ 19 ^)^, can contribute to the rational use of resources. Although identification is not simple, it is essential for patient safety and survival. In this sense, observation of patients with complications in subgroups may contribute to the adoption of preventive rather than reactive therapies. 

Severe circulatory complications were the most important factor, increasing the risk of death by seven times. In this study, three conditions portray the set of serious circulatory complications: cardiorespiratory arrest (CRA); deep vein thrombosis and acute myocardial infarction (AMI). These complications in traumatized patients, even fewer incidents, can be lethal. In the analysis of complications of ICU trauma patients at level 1 and 2 trauma centers of the largest trauma database in the USA in 2013, CRA (OR-9.5) was identified as one of the main factors that increased chance of death^(^ 19 ^)^.

Although, in this study, it is not possible to establish whether circulatory complications occur before or after ICU admission, or even if they have a direct relationship with comorbidities, trauma can become a decisive factor for its triggering by making the individual fragile. and by exposing you to excessive interventions and procedures. The development of complications during ICU trauma hospitalization may be a clinical factor to safely and carefully determine the outcome of intensive care, such as death or longer hospital stay^(^ 19 ^)^.

Another factor contributing to mortality in traumatized patients is hemodynamic instability and, in such cases, adequate tissue perfusion with early administration of crystalloid fluids should be ensured. Vasoactive drugs may be transiently required in the presence of life-threatening hypotension^(^ 34 ^))^ and early use may limit organ hypoperfusion and prevent multiple failure^(^ 28 ^)^. However, evidence identified in a systematic review of the early use of vasopressors after traumatic injury highlights that, in addition to the benefits, some damage from vasopressor therapy in the early phase of trauma is also reported, such as the risk of bleeding, coagulopathy, compartment syndrome, and surgical complications^(^ 34 ^)^.

In this study, the use of vasopressors remained independently associated with death. Similarly, research that tracked trauma-level inpatients in the US between 2011 and 2016 who used red blood cell transfusion upon admission found that mortality gradually increases with increased use of vasoactive agents^(^ 35 ^)^. Even in the face of controversies regarding the use of vasoactive drugs for traumatized patients, the admission of these patients to an intensive setting allows careful and continuous management with instant monitoring of their vital functions. 

Despite the heterogeneity of trauma patients with different respiratory needs, a large proportion of patients require mechanical ventilation due to acute respiratory failure (ARF)^(^ 36 ^)^, as is the case of the population studied. In this regard, the use of ventilatory support depends on the severity of respiratory dysfunction, impairment of gas exchange, associated trauma, and the feasibility of using noninvasive mechanical ventilation (NIV)^(^ 36 ^)^ or for airway protection and prevention of secondary brain injury^(^ 37 ^)^ and other conditions such as hemorrhagic shock and multiple organ damage^(^ 38 ^)^. Thus, regardless of the need for mechanical ventilation, trauma patients share common ICU care. In this study, mechanical ventilation, regardless of the justification for its use, increased the probability of death fivefold. 

With the exception of patients intubated for airway protection, there are alternatives to avoid mechanical ventilation and reduce associated complications^(^ 36 ^)^. To prevent complications and death, the use of noninvasive mechanical ventilation^(^ 36 ^)^ and pressure-controlled ventilation combined with spontaneous breathing^(^ 39 ^)^ may be alternatives to mechanical ventilation.

Renal dysfunction during the first 24 hours was a risk factor for ICU death in the cohort analyzed. Excessive and inadequate immune response to trauma is known to result in multiple organ dysfunction and cell injury, which in turn may lead to death^(^ 40 ^)^. The identification by the intensive care team of patients with this dysfunction may be an action to prevent death and other more severe renal complications that may develop during ICU stay.

Thus, such identification becomes important for clinical practice, since renal dysfunction presents physiological responses to the lesion that may be reversible, unlike renal failure ^(^ 41 ^)^, and the first minutes or hours after trauma are critical to an adequate immune response. A study in London showed that immune system function in severely traumatized patients is associated with the development of organ dysfunction in a hyperacute phase (up to two hours)^(^ 40 ^)^. This pathophysiological knowledge becomes important for efficient treatment, especially in the ICU, because, due to the complexity in PHC and transport logistics, the time until a definitive assistance can contribute to the development of organic dysfunctions such as renal.

It is known that some factors may worsen the prognosis of patients with posttraumatic kidney injury such as: inadequate resuscitation; hypotension; diabetes; hypertension; pre-existing renal failure; sepsis and nephrotoxins^(^ 42 ^)^. Just as trauma is a major diagnosis of ICU admission in developing countries, the epidemiology of posttraumatic renal dysfunction becomes a major complication, as in the study by Sobral - CE, which found an incidence of 32.9% of acute kidney injury, and a profile of older diabetic patients who stayed longer in the ICU, who had higher APACHE and often used mechanical ventilation and vasopressors^(^ 8 ^)^.

Infection prevalence is an indicator of outcome quality and prevention is part of an interdisciplinary and inter-professional effort to improve ICU car^(^ 39 ^)^. Therefore, the blood culture test should be performed at the time of admission to the ICU, as it contributes to the monitoring and prevention of infections and indicates the treatment with decision and choice of appropriate antibiotic^(^ 39 ^)^. The use of appropriate microbiological tests is one of the indicators to control and prevent ICU infections which, coupled with clinical signs such as level of consciousness, respiratory rate, systolic blood pressure and organ failure assessment, can monitor infection, as indicated by the German Society of Intensive Care Medicine^(^ 39 ^)^. 

Performing a blood culture test on ICU admission is a timely measure^(^ 43 ^)^ especially for patients predisposed to stay in the unit for a long time. In this study, the risk factor for death was the non-performance of blood culture at the time of ICU admission. Failure to perform the test hinders both the assessment of care and the interpretation of quality indicators established to monitor and control the occurrence of healthcare-associated infections^(^ 44 ^)^. Empirical antimicrobial therapy, without identifying the bacterium that causes the infection and which is based on the patient’s symptoms, tends to produce the opposite result as it may result in increased duration of antibiotic treatment, length of hospital stay, resistance to multiple drugs and mortality rate in critically ill patients^(^ 45 ^)^.

Measuring disease severity is critical to drive care, and one of the most commonly used routine indicators in an intensive care setting is APACHE II, which has been shown to be sufficient to predict death in trauma patients^(^ 32 ^,^ 46 ^)^. The results of this study also demonstrated the association of patient severity as measured by APACHE II and death. Although the use of APACHE II is time consuming and costly, the index estimates the prognosis for ICU admission and may be appropriate for assessing and monitoring trauma patients by identifying abnormal physiology^(^ 46 ^)^ and reducing preventable deaths^(^ 32 ^)^. 

Regardless of the type of trauma and the place where the traumatized will be assisted, it is considered one of the health problems with the greatest impact on the health and economy of contemporary society. Although prevention of morbidity and mortality remains a major challenge in developing countries and at all levels of care, in Brazil, this challenge should focus on preventing accidents and violence through behavioral changes through information campaigns against alcohol and drug use, gun control, fall prevention strategies and speed limit surveillance, secondary prevention, in order to reduce the severity of trauma through the use of seat belts, helmets, child seats, among other measures^(^ 47 ^)^.

In the event of trauma in the country, PHC teams prepared for the first care are needed, as well as efficient structures to stabilize the patient, such as the UPA and the hospital emergency room. If the individual needs intensive care, complication prevention strategies can impact survival, even in the face of structural and human resource constraints. Therefore, traumatic injuries are not managed alone by a single professional and in a single place of care, but rather, interprofessionally, and with this, the ICU nurse stands out.

Data from this cohort, analyzed at hierarchical levels, identified predictors of death. Although factors at the distal and intermediate levels are important in determining death for clinical practice, it is those at the proximal level that help researchers and health professionals to administer direct patient care. These factors reinforce the daily challenge of nurses to guide their intensive practice based on evidence, but also based on clinical experience and patient values. In this sense, these professionals should be equipped to know the epidemiology of trauma, the natural history, the various factors involved and the strategies and possible interventions to impact the incidence (primary prevention) on its severity (secondary prevention) or its consequences (tertiary prevention).

The results may also be useful for future epidemiological and clinical studies as they consider complex trauma-determining variables not explored in this study, such as traumas exposure characteristics related to human, social, health, occupational, political and cultural behavior. 

Despite the relevant data obtained in this study, limitations should be highlighted, such as the collection in medical records, which may not contain all records, resulting in exclusions of individuals, and the fact that the study was conducted in a single ICU in a region of a state in Brazil, as it does not allow the generalization of the results. 

## Conclusion

This research identified an ICU trauma mortality rate of 28.2% and risk factors that are useful in composing a clinical profile of trauma patients admitted to the ICU. The hierarchical determination of some factors over others, especially those near the proximal level of death, such as circulatory complications, use of vasoactive drugs and mechanical ventilation, the occurrence of renal dysfunction in the first 24 hours, elevated APACHE blood culture examination at admission showed that, for these patients undergoing intensive care, indicators of qualified hospital care have priority in the prevention of clinical complications.
